# Effects of mental fatigue on basketball-specific decision-making in basketball players of different skill levels: the mediating role of attentional control

**DOI:** 10.3389/fpsyg.2026.1832492

**Published:** 2026-05-22

**Authors:** Yifan Hu, Qiulin Wang

**Affiliations:** College of Physical Education, Yangzhou University, Yangzhou, Jiangsu, China

**Keywords:** attentional control, basketball, mediation, mental fatigue, sport decision-making

## Abstract

**Objective:**

To investigate the effects of mental fatigue on sport decision-making performance in basketball players with different skill levels and to examine whether attentional control mediates this association.

**Methods:**

Forty-two male basketball players were recruited, including 21 higher-skilled players and 21 lower-skilled players. Mental fatigue was induced using a Stroop task. Before and after fatigue induction, participants completed a basketball-specific video-based decision-making task and the Eriksen Flanker task. Repeated-measures analyses were conducted to examine the effects of mental fatigue and group differences, and bootstrap mediation analyses were used to test the mediating role of attentional control. Key variables were subjected to data cleaning and robustness checks.

**Results:**

After mental fatigue induction, basketball-specific decision accuracy significantly decreased, *F*(1, 40) = 132.789, *p* < 0.001, η*_p_*^2^ = 0.769, whereas decision reaction time significantly increased, *F*(1, 40) = 98.445, *p* < 0.001, η*_p_*^2^ = 0.711. The higher-skilled group outperformed the lower-skilled group in both decision accuracy, *F*(1, 40) = 49.200, *p* < 0.001, η*_p_*^2^ = 0.552, and decision reaction time, *F*(1, 40) = 16.845, *p* < 0.001, η*_p_*^2^ = 0.296. In the Flanker task, the RT conflict effect significantly increased after mental fatigue induction, *F*(1, 40) = 146.091, *p* < 0.001, η*_p_*^2^ = 0.785. Mediation analysis showed that attentional control change partially mediated the relationship between subjective fatigue rating change and prolonged basketball-specific decision reaction time, indirect effect = 4.966, 95% BootCI (1.621, 8.513). This mediation pattern was further supported by the residualized change-score robustness analysis, Effect = 5.494, 95% BootCI (1.607, 9.774).

**Conclusion:**

Mental fatigue impairs decision-making performance in basketball players, and the magnitude of this impairment varies by skill level under mental fatigue. Attentional control partially mediated the association between subjective fatigue rating change and basketball-specific decision reaction time, particularly with respect to response-time outcomes.

## 1 Introduction

As a high-intensity open-skill sport (Deng et al., 2025), basketball is characterized by dynamic and uncertain competitive environments in which athletes must rapidly extract critical information and make accurate decisions under tight time constraints. Sport decision-making (Zhao, 2025) refers to the cognitive process by which athletes use environmental cues and prior experience to rapidly select and execute an optimal course of action during competition. In basketball, athletes’ decision-making performance is often an important determinant of match outcomes (Song et al., 2024). This is particularly evident in elite sport, where millisecond-level differences in decision speed may influence competitive outcomes; therefore, decision-making is commonly regarded as a key indicator of expertise (Jin et al., 2023).

During high-intensity training and competition, athletes are often required to maintain a heightened level of attention over extended periods while continuously engaging in complex tactical processing and sport-specific decision-making. Such sustained cognitive demands may readily lead to mental fatigue (Qian, 2024). Unlike peripheral muscular fatigue, mental fatigue is a subjective state of psychobiological exhaustion caused by prolonged cognitive activity and is typically characterized by attentional lapses, delayed responses, and reduced motivation (Mehta and Parasuraman, 2014). In basketball, performance unfolds in an environment characterized by continuous information-processing demands and substantial time pressure. Players must continuously monitor the environment, make tactical judgments, and select appropriate actions; consequently, their decision-making performance may be particularly vulnerable to the effects of mental fatigue (Bian et al., 2025). In recent years, increasing attention has been directed toward the role of mental fatigue in high-level team-sport performance contexts (Wu et al., 2024). Previous research has suggested that, in cognitively demanding contexts such as cumulative training and competition demands, tactical execution, and in-game decision-making, mental fatigue may constitute an important constraint on performance in elite team sports (Russell et al., 2019). On this basis, it may be reasonably inferred that decision-making performance in basketball players is particularly sensitive to mental fatigue.

However, simply demonstrating that mental fatigue affects basketball decision-making is not sufficient; a more critical question concerns the cognitive processes through which this effect occurs. Previous research has generally suggested that mental fatigue first impairs the processing efficiency of cognitive control (Ma and Ren, 2025). In basketball, players must continuously maintain goal-relevant information, inhibit irrelevant stimuli, and rapidly complete response selection under multiple sources of interference, all of which depend heavily on attentional control. According to attentional control theory, adverse psychological states weaken goal-directed top-down attentional control, making individuals more susceptible to stimulus-driven bottom-up processing (Ozturker et al., 2025). Although this theory was originally developed primarily to explain the effects of anxiety on cognitive performance, its central assumption that processing efficiency is impaired before performance effectiveness provides a useful framework for understanding fatigue-related reductions in cognitive control. Basketball-specific decision-making requires players to inhibit irrelevant cues, maintain current tactical goals, and select optimal actions rapidly in complex offensive and defensive contexts; therefore, attentional control may serve as a key cognitive mechanism linking mental fatigue to basketball-specific decision-making performance (Cao et al., 2025). Attentional control refers to the ability to voluntarily allocate attentional resources, maintain goal-directed processing, and suppress interference from irrelevant stimuli in the presence of competing or conflicting information (Han, 2024). Although prior studies have suggested that attentional control is important for efficient decision-making in basketball (Zhao, 2024), direct empirical evidence remains limited regarding whether mental fatigue influences basketball-specific decision-making through impaired attentional control.

At the same time, it remains unclear whether this potential pathway operates differently across athletes with different skill levels. Because athletes at different skill levels differ in situation representation, key-cue extraction, and strategy retrieval, the effects of mental fatigue on attentional control and decision-making may differ across groups. Previous studies indicate that higher-level athletes generally exhibit more efficient processing structures and more stable strategy repertoires in key-cue extraction, situation representation, and pattern recognition (Chu and Wang, 2024). This suggests that the extent of attentional control impairment and decision-making impairment under mental fatigue may vary across athletes of different skill levels. However, prior research has largely focused on the direct effects of mental fatigue on physical or technical performance, and direct evidence remains limited regarding whether the relationship between attentional control and decision-making differs across basketball players with different skill levels under mentally fatigued conditions (Van Cutsem et al., 2017). Against this background, the present study recruited basketball players with different skill levels, induced mental fatigue using a cognitive task, and employed an attentional control paradigm alongside a basketball decision-making task to investigate the effects of mental fatigue on decision-making performance, with particular emphasis on examining the mediating role of attentional control. In the present study, skill level was classified based on official athlete grade, years of systematic training, and competitive level. The higher-skilled group consisted of basketball players who held National First-Class Athlete certification and competed in national-level collegiate basketball competitions, whereas the lower-skilled group consisted of players who held National Second-Class Athlete certification and competed primarily in university-level competitions. Importantly, the lower-skilled group should not be interpreted as truly inexperienced novices; rather, these participants were trained basketball players whose competitive level and competition experience were lower than those of the higher-skilled group. Accordingly, the following hypotheses were proposed: H1: Mental fatigue would significantly reduce basketball players’ decision-making performance. H2: Compared with the lower-skilled group, the higher-skilled group demonstrated better and more stable decision-making performance under mental fatigue. H3: Higher levels of mental fatigue would be associated with reduced attentional control. H4: Attentional control would mediate the relationship between mental fatigue and decision-making performance. RQ5: Does skill level moderate the indirect effect of mental fatigue on decision-making through attentional control?

## 2 Participants and methods

### 2.1 Participants

Because few prior studies employed a design closely comparable to that of the present study, obtaining a stable and directly transferable *a priori* effect size estimate was challenging. Therefore, sample size estimation was conducted using G*Power 3.1.9.4 (Faul et al., 2009), using Cohen’s *f* as the effect size index for the ANOVA. Following conventional criteria for a medium effect in the social and behavioral sciences, *f* = 0.25 was used as the primary effect size estimate (Cohen, 1988), with *α* = 0.05 and statistical power set at 0.80. Power analysis indicated that a minimum sample of 34 participants was required to test the main effects. To account for potential attrition, the planned sample size was increased by approximately 20%, yielding a final recruitment target of 42 participants. Participants were assigned to two groups according to skill level. The higher-skilled group consisted of 21 collegiate basketball players who held National First-Class Athlete certification, had received long-term systematic basketball-specific training, and competed in national-level collegiate basketball competitions such as the Chinese University Basketball Association (CUBA). The lower-skilled group consisted of 21 players who held National Second-Class Athlete certification, had also received systematic basketball training, and participated in university-level basketball competitions, but had not competed in national-level competitions such as CUBA (see Table 1).

**Table 1:** Participant characteristics.

Group	*n*	Competitive level	Training experience (years)	Age (years)
Higher-skilled group	21	National Level I basketball athletes	9.52 ± 0.92	20.19 ± 1.28
Lower-skilled group	21	National Level II basketball athletes	3.95 ± 0.86	19.95 ± 0.58

Values are mean ± SD.

Group classification was based not solely on years of training, but on a combination of indicators, including official athletic classification, competition level, and systematic training background. This grouping strategy was grounded in a formal institutional framework (General Administration of Sport of China, 2024). Under China’s official athlete classification system, established by the General Administration of Sport, National First-Class Athletes generally represent a higher competitive standard and are required to meet prescribed criteria in national or higher-level competitions. By contrast, National Second-Class Athletes generally refer to athletes who have received systematic training and attained the required sport-specific competencies, yet whose competition level and performance standards are typically lower than those of National First-Class Athletes. All participants were right-handed and had normal vision. None had previously completed video-based choice tasks or sport-decision training; none reported excessive fatigue prior to the experiment; and all refrained from caffeine intake for at least 3 h before testing. All participants met the inclusion criteria and provided informed consent to participate in the study.

### 2.2 Experimental design

The present study employed a 2 (mental fatigue: pre-induction vs. post-induction) × 2 (group: higher-skilled vs. lower-skilled) mixed factorial design, with mental fatigue treated as a within-subject factor and group as a between-subject factor. Decision-making accuracy and reaction time were specified as the outcome variables. Attentional control was specified as the mediating variable and operationalized as indexed by both the conflict effect derived from the Eriksen Flanker task (Eriksen and Eriksen, 1974) and the pre- to post-induction change in that effect. Mental fatigue was experimentally induced using a Stroop color-word interference task (Stroop, 1992). To verify the specificity of the fatigue induction procedure, a duration-matched neutral-video control condition was included. This control condition was used only for the subjective manipulation check, to preliminarily determine whether changes in subjective fatigue ratings were primarily attributable to the cognitive load induced by the Stroop task rather than merely to the passage of time. It was not included in the subsequent primary mechanistic model analyses (Bian et al., 2025). All experimental tasks were presented and recorded using E-Prime 3.0.

### 2.3 Apparatus

The experiment was performed using a Lenovo ThinkBook 16 laptop equipped with a 16-inch display (1,920 × 1,080 resolution) and a 120 Hz refresh rate. All tasks were presented, and responses were recorded using E-Prime 3.0.

### 2.4 Materials

Decision videos: To ensure ecological and technical representativeness, all decision-making video clips were selected from official Chinese Basketball Association (CBA) games. To balance visual clarity, tactical consistency, and recency, only clips from recent seasons were considered, with recordings from the 2024 season ultimately selected as the source material. Following established procedures for constructing video-based decision-making stimuli and taking into account recommendations from athletes and experts (Miao and Chi, 2017), each clip began at the start of an offensive possession and was occluded at the critical moment just before the ball handler executed the next action. Following occlusion, participants responded by keypress, and the system automatically recorded reaction time and decision accuracy. In the present study, the actual choice made by the ball handler during the game was not treated as the correct answer; instead, it served only as an ecological reference, and the final scoring criterion was determined on the basis of expert evaluation. Specifically, 72 candidate video clips were initially selected. Two basketball referees and three university basketball coaches independently judged the optimal decision at the freeze-frame moment in each clip, with the response options including passing, driving, and shooting. During the expert evaluation, the panel considered offensive and defensive positioning, passing lanes, driving space, shooting opportunities, defensive pressure, teammate positioning, and the executability of each option under the specific spatiotemporal constraints of the game context. To assess inter-rater agreement, Fleiss’ *κ* was calculated for the initial expert ratings. The results indicated a high level of agreement among the experts, Fleiss’ κ = 0.89. Clips were excluded if expert judgments showed clear disagreement, the decision point was ambiguous, key contextual information was occluded, offensive or defensive positioning was difficult to identify, or the expert-validated optimal option was judged to be insufficiently executable in the specific game context. The remaining clips were then reviewed by one referee and two university basketball coaches, and only clips with consistent expert agreement and clearly defined decision types were retained. This process resulted in 60 eligible clips, with a balanced distribution of passing, driving, and shooting decisions. Participants’ responses were scored as correct when they matched the expert-validated optimal option. Thus, in the present study, decision accuracy refers to the degree of correspondence between participants’ choices and the expert-validated context-specific optimal decision, rather than correspondence with the actual decision made by the ball handler in the original game clip.

### 2.5 Experimental procedure

#### 2.5.1 E-Prime–based sport decision-making task

**Figure 1:**
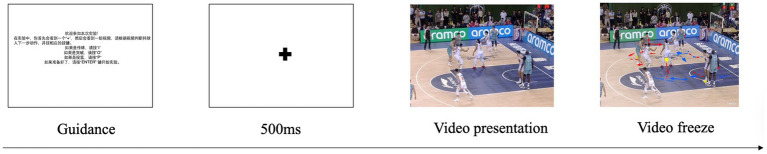
E-Prime sport decision-making task flowchart.

E-Prime 3.0 was used to present the basketball decision-making task and record decision-making accuracy and response time. According to the on-screen instructions, the keys I, O, and P corresponded to pass, drive, and shoot, respectively. Each trial began with a central fixation cross presented for 500 ms, followed by the presentation of an offensive video clip. Participants were instructed to judge the ball handler’s next action and respond using the corresponding key. The task consisted of a practice phase (5 trials) and a formal test phase. After familiarizing themselves with the task requirements, participants pressed ENTER to begin the formal test phase. The formal task consisted of two blocks, each containing 30 trials, with decision scenarios and task difficulty matched across blocks. Trial order was randomized within each block. The total task duration was approximately 10 min. To control for practice effects, different video scenarios were used for the pre- and post-tests. The procedure is shown in Figure 1.

#### 2.5.2 Stroop color–word interference task

**Figure 2:**
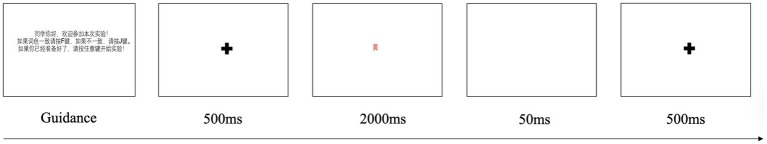
Stroop task flowchart.

Building on prior research, a Stroop color–word interference task was used in the present study to induce mental fatigue (Van Cutsem et al., 2017). A pilot study was conducted to verify the effectiveness of a 30-min continuous Stroop task, which confirmed that this protocol was sufficient to induce mental fatigue. Following presentation of the task instructions, each trial began with a 500-ms fixation cross. Participants were instructed to respond as quickly as possible to the ink color while ignoring the word meaning. Participants responded to indicate whether the word meaning and ink color were congruent (F) or incongruent (J). The task lasted 30 min without breaks. The practice phase consisted of 32 trials, and the formal task consisted of a single block of 960 trials presented without interruption. A visual analogue scale (VAS) was administered before and after the mental fatigue induction procedure as the primary manipulation check to assess subjective fatigue (Daneshgar-Pironneau et al., 2025). In addition, objective behavioral indices during the Stroop task (response time and accuracy) were recorded as supplementary indicators of the success of the manipulation. The procedure is shown in Figure 2.

#### 2.5.3 Eriksen flanker task

**Figure 3:**
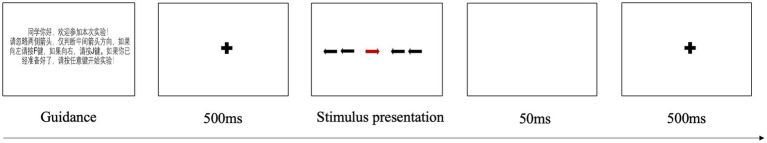
Flanker task flowchart.

Attentional control was assessed using the Eriksen Flanker paradigm to measure executive attentional functions, particularly interference inhibition and conflict resolution (Eriksen and Eriksen, 1974). The task was presented using E-Prime 3.0, and performance data were recorded automatically. The stimuli consisted of a sequence of five arrows, with the central arrow designated as the target stimulus and the flanking arrows designated as distractors. In the congruent condition, the flankers were oriented in the same direction as the central arrow, whereas in the incongruent condition, the flankers were oriented in the opposite direction. Participants were instructed to ignore the flanking arrows and respond only to the direction of the central arrow by pressing the corresponding key (F = left; J = right). Each trial began with a 500-ms fixation cross, followed by the simultaneous presentation of the target and distractor stimuli. Participants responded via keypress, and the system automatically recorded reaction time and accuracy. The formal task consisted of two blocks, each containing 40 trials. Congruent and incongruent trials were presented in a 1:1 ratio and randomized order, with a 30-s rest interval between blocks. Attentional control was quantified using the conflict effect, which was calculated as follows: RT conflict effect = mean RT in the incongruent condition minus mean RT in the congruent condition; accuracy conflict effect = mean accuracy in the congruent condition minus mean accuracy in the incongruent condition (Van der Lubbe et al., 2025). The experimental procedure is shown in Figure 3.

Although the Flanker task is not specific to basketball decision-making, it has been extensively employed to assess interference inhibition and conflict control in selective attention, and standardized cognitive assessment systems also identify it as a measure of inhibitory control and attentional functioning (Lee and Pitt, 2024). Given the context-dependent nature of decision-making in team sports, the present study combined the Flanker task with a basketball-specific decision-making task: the former assessed general cognitive mechanisms, while the latter captured decision-making performance in a sport-specific context (Zhu et al., 2024). The combination of these two tasks enabled the assessment of both the underlying mechanisms of attentional control and its behavioral manifestation in basketball decision-making (Cao et al., 2025).

### 2.6 Experimental procedure and control of variables

To reduce the potential influence of learning effects and cumulative task demands associated with the pretest on the experimental results, a familiarization session was conducted prior to the main experiment. This session enabled participants to familiarize themselves with the procedures, response requirements, and pacing of both the Flanker task and the video-based decision-making task. Data from the familiarization session were not included in the subsequent statistical analyses.

Upon arriving at the laboratory individually, participants first completed a health screening, completed a demographic questionnaire, provided informed consent, and completed task practice trials. The visual analogue scale (VAS) was then administered to assess baseline subjective fatigue. Participants then completed the Flanker task followed by the basketball-specific decision-making task to obtain baseline measures of attentional control and sport decision-making before fatigue induction. In the present study, the order of the Flanker task and the basketball-specific decision-making task was kept constant across all participants, with the Flanker task administered before the decision-making task. This fixed order was used to maintain procedural consistency and to ensure that attentional control was assessed before sport decision-making, thereby aligning with the hypothesized theoretical pathway in which attentional control influences sport decision-making. The Flanker task was relatively short and served primarily as a standardized measure of attentional control; its cognitive load was substantially lower than that of the 30-min sustained Stroop fatigue-induction task. The same task order was used for all participants to ensure consistency across groups. A 30-min sustained Stroop task was then administered to induce mental fatigue. Following the induction procedure, the VAS was administered again, after which participants completed the Flanker task and the sport decision-making task to obtain post-induction measures of attentional control and decision-making performance. To verify the specificity of the fatigue induction procedure, a duration-matched neutral-video control condition was also included. In the control condition, participants viewed a 30-min neutral video that did not involve high cognitive demands, strong emotional stimulation, or basketball-specific content. The VAS was administered before and after the viewing period. This control condition was used solely for the subjective manipulation check and was not included in the main mechanistic model analyses.

To enhance internal validity, potential confounding factors, including the experimental environment, testing time window, task instructions, sleep status, and caffeine intake, were standardized as far as possible. Participants’ training background was also recorded for sample characterization and subsequent statistical analyses.

### 2.7 Data processing

Decision accuracy and reaction time data were extracted using E-Prime 3.0. Trials with omitted responses or incorrect trials were excluded, as were trials with RTs shorter than 200 ms. For the remaining trials, extreme values were removed using an individual mean ± 3 SD criterion, and mean values were subsequently computed for each participant (see Table 2). Descriptive results showed that the proportions of trials excluded due to RTs shorter than 200 ms and ±3 SD extreme values were low across groups and conditions. No group or time condition showed an obviously abnormal exclusion rate, suggesting that extreme RT trimming did not introduce substantial systematic bias into the main results. Because the study focused on state changes induced by the mental fatigue manipulation and the mechanisms through which these changes influenced sport decision-making performance, pre–post difference scores were used in the mediation model to represent the magnitude of change in each variable. In the context of fatigue-induced change, difference scores more directly capture within-individual changes from pre- to post-manipulation (Gu et al., 2018). SPSS 27.0 was used for descriptive statistics and normality testing of mean decision accuracy and reaction time. Mediation effects were examined using Model 4 in PROCESS v4.1, with difference scores entered for each variable (Preacher and Hayes, 2008). Bootstrap resampling (5,000 iterations) was used to estimate the indirect effect and its 95% confidence interval; mediation was considered significant when the confidence interval did not include zero. In the exploratory analyses, PROCESS Model 7 was used to examine the moderated mediation effect of skill level. Because the moderated mediation analysis involved testing an interaction effect, which typically requires a larger sample size, a *post hoc* sensitivity analysis was conducted using G*Power 3.1 to evaluate the ability of the current sample to detect the interaction term. The parameters were set as follows: *α* = 0.05, power (1 − *β*) = 0.80, total sample size *N* = 42, number of tested predictors = 1, and total number of predictors = 4. It should be noted that the sample size estimation in the present study was based primarily on repeated-measures ANOVA rather than being specifically powered for the moderated mediation model. Therefore, the moderated mediation analysis involving skill level was reported as an exploratory analysis.

**Table 2:** Trial exclusion information for reaction-time analyses.

Task	Group	Time	Total trials	Error/omission exclusion rate	RT < 200 ms exclusion rate	±3 SD exclusion rate	Total exclusion rate
Basketball-specific decision-making task	Higher-skilled group	Pre-fatigue	1,260	33.10%	0.16%	0.95%	34.21%
Basketball-specific decision-making task	Higher-skilled group	Post-fatigue	1,260	40.95%	0.24%	1.19%	42.38%
Basketball-specific decision-making task	Lower-skilled group	Pre-fatigue	1,260	46.19%	0.32%	1.11%	47.62%
Basketball-specific decision-making task	Lower-skilled group	Post-fatigue	1,260	59.05%	0.40%	1.43%	60.87%
Flanker task	Higher-skilled group	Pre-fatigue	1,680	2.20%	0.06%	1.07%	3.33%
Flanker task	Higher-skilled group	Post-fatigue	1,680	2.86%	0.12%	1.31%	4.29%
Flanker task	Lower-skilled group	Pre-fatigue	1,680	2.98%	0.12%	1.19%	4.29%
Flanker task	Lower-skilled group	Post-fatigue	1,680	3.99%	0.18%	1.49%	5.65%

RT, reaction time; SD, standard deviation. Error/omission trials were excluded before RT-based trimming. Extreme RT values were identified using a participant-specific mean ± 3 SD criterion within each task and time point.

To address concerns that difference scores may involve measurement error and reduced reliability, residualized change scores were further used as a supplementary robustness check. Specifically, the post-induction Flanker RT conflict effect was regressed on the pre-induction Flanker RT conflict effect, and the unstandardized residual was extracted as a baseline-adjusted indicator of attentional control change. This residualized change score was then entered into the mediation model in place of the simple difference score to examine the robustness of the main mediation results. In addition, given the between-group difference in years of training, supplementary models were conducted with years of training and age entered as covariates to examine whether the main findings were influenced by differences in training experience and age.

## 3 Results

### 3.1 Manipulation check for mental fatigue induction

To examine whether the 30-min sustained Stroop task effectively induced a mental fatigue state, subjective fatigue experience was assessed using the VAS before and after the task. Changes in reaction time and accuracy during the Stroop task were also used as supplementary behavioral manipulation-check indices. It should be noted that VAS scores primarily reflect participants’ subjective experience of fatigue, whereas mental fatigue, as a psychological state induced by prolonged cognitive activity, is not equivalent to subjective fatigue ratings themselves. Therefore, in the present study, VAS scores were used as a subjective manipulation-check indicator of mental fatigue induction, and behavioral changes during the Stroop task were used as complementary evidence for the effectiveness of the induction. The results revealed a significant increase in VAS scores after the intervention compared with before the intervention under the mental fatigue induction condition, and the magnitude of change was significantly greater than that observed in the control condition (*p* < 0.001), indicating that the sustained Stroop task effectively induced mental fatigue. An additional objective manipulation check was conducted based on Stroop task performance data, with the formal task segmented into the first and last 10-min intervals. The results indicated that the RT interference effect during the last 10-min interval was significantly greater than during the first 10-min interval [*t*(41) = 5.87, *p* < 0.001, *d* = 0.91]. Similarly, the accuracy interference effect during the last 10-min interval was significantly greater than during the first 10-min interval [*t*(41) = 6.75, *p* < 0.001, *d* = 1.04]. The subjective VAS scores and the behavioral indicators of the Stroop process jointly suggest that the mental fatigue induction procedure used in this study was effective (see Tables 3, 4).

**Table 3:** Descriptive statistics for VAS scores before and after mental fatigue induction (M ± SD).

Group	*n*	Pre-test under the mental fatigue condition	Post-test under the mental fatigue condition	Pre-test under the control condition	Post-test under the control condition
Higher-skilled group	21	29.05 ± 1.59	48.57 ± 3.89	28.29 ± 2.92	34.95 ± 4.23
Lower-skilled group	21	31.62 ± 2.17	54.24 ± 5.29	30.14 ± 3.02	37.67 ± 3.03

VAS, visual analogue scale, data are presented as mean ± standard deviation (M ± SD).

**Table 4:** Descriptive statistics for RT and ACC before and after the Stroop task (M ± SD).

Group	*n*	RT during the first 10 min (ms)	ACC during the first 10 min	RT during the last 10 min (ms)	ACC during the last 10 min
Higher-skilled group	21	940.50 ± 101.67	0.93 ± 0.25	1076.43 ± 122.67	0.87 ± 0.33
Lower-skilled group	21	974.58 ± 99.52	0.92 ± 0.03	1104.55 ± 120.78	0.85 ± 0.05

RT, reaction time; ACC, accuracy. Data are presented as mean ± standard deviation (M ± SD).

### 3.2 Effects of mental fatigue on sport decision-making in basketball players across skill levels

#### 3.2.1 Repeated-measures ANOVA results for decision accuracy

**Figure 4:**
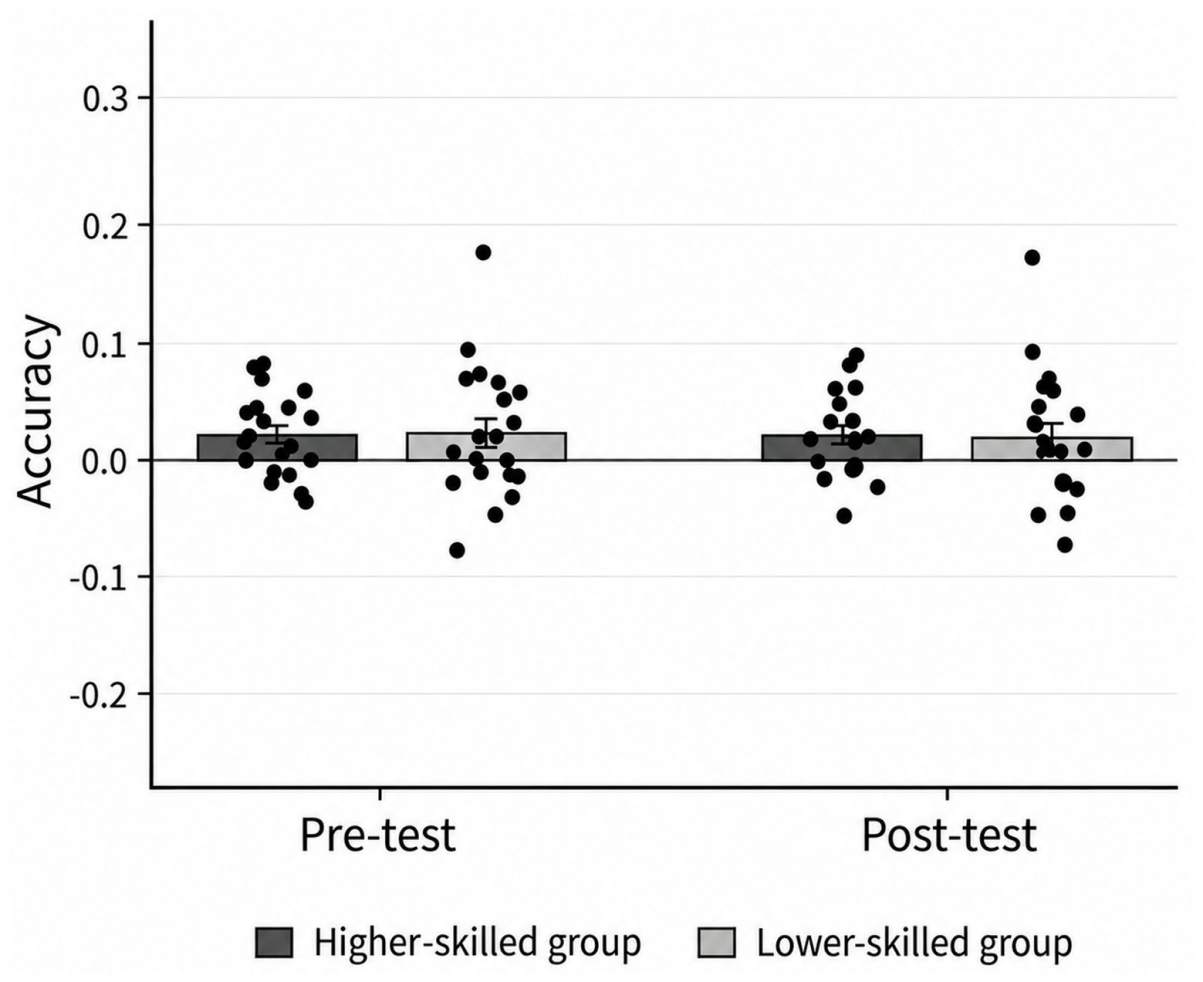
Comparison of decision accuracy before and after fatigue among different groups. *p < 0.05, **p < 0.01, ***p < 0.001.

A repeated-measures ANOVA was conducted with decision accuracy as the dependent variable and time (pre vs. post) and group (higher-skilled vs. lower-skilled) as factors. The results (Table 5) revealed a significant main effect of time, *F*(1, 40) = 132.789, *p* < 0.001, η*_p_*^2^ = 0.769, 95% CI (0.622, 0.837), with decision accuracy being significantly higher before induction than after induction. The main effect of group was also significant, *F*(1, 40) = 49.200, *p* < 0.001, η*_p_*^2^ = 0.552, 95% CI (0.325, 0.681), indicating that decision accuracy was significantly higher in the higher-skilled group than in the lower-skilled group. The time × group interaction was significant, *F*(1, 40) = 7.737, *p* < 0.05, η*_p_*^2^ = 0.162, 95% CI (0.012, 0.356), indicating that the magnitude of the pre-to-post change in accuracy differed between groups. Follow-up simple effects analyses showed that decision accuracy decreased significantly from pre- to post-induction in both groups, ps < 0.01. As shown in Figure 4, the reduction in decision accuracy was greater in the lower-skilled group than in the higher-skilled group.

**Table 5:** Descriptive statistics for decision accuracy before and after mental fatigue induction in basketball players of different skill levels (%, M ± SD).

Group	*n*	Pre-fatigue	Post-fatigue
Higher-skilled group	21	66.90 ± 6.07	59.04 ± 6.82
Lower-skilled group	21	53.81 ± 9.87	40.95 ± 7.35

#### 3.2.2 Repeated-measures ANOVA results for decision response time

**Figure 5:**
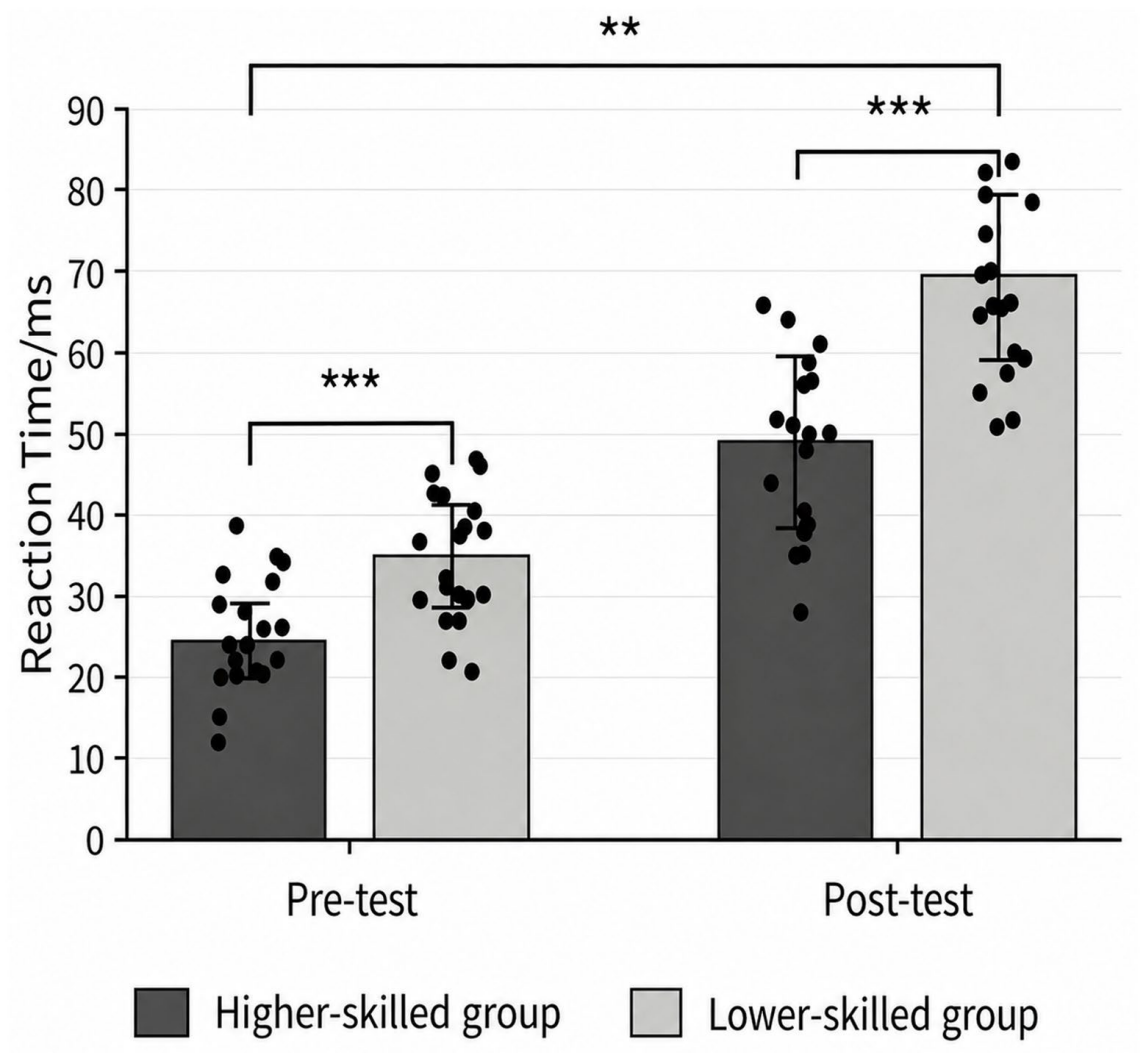
Comparison of decision reaction time before and after fatigue among different groups. *p < 0.05, **p < 0.01, ***p < 0.001.

A repeated-measures ANOVA was conducted with decision-making reaction time (RT) as the dependent variable and time (pre-fatigue vs. post-fatigue) and group (higher-skilled vs. lower-skilled) as factors. The results (Table 6) revealed a significant main effect of time, *F*(1, 40) = 98.445, *p* < 0.001, η*_p_*^2^ = 0.711, 95% CI (0.536, 0.796), indicating that decision-making RT was significantly longer after fatigue induction than before fatigue induction. The main effect of group was also significant, *F*(1, 40) = 16.845, *p* < 0.001, η*_p_*^2^ = 0.296, 95% CI (0.081, 0.481), indicating that RTs were significantly longer in the lower-skilled group than in the higher-skilled group. The time × group interaction was not significant, *F*(1, 40) = 1.834, *p* = 0.183, η*_p_*^2^ = 0.044, 95% CI (0.000, 0.217), indicating that the magnitude of the pre-to-post change in RT did not differ significantly between groups. Accordingly, the fatigue manipulation did not produce a differential pre–post change in RT across groups (see Figure 5).

**Table 6:** Descriptive statistics for decision reaction time before and after mental fatigue induction in basketball players of different skill levels (ms, M ± SD).

Group	*n*	Pre-fatigue	Post-fatigue
Higher-skilled group	21	1501.13 ± 478.64	1866.45 ± 583.41
Lower-skilled group	21	2034.37 ± 436.61	2515.20 ± 432.55

### 3.3 Effects of mental fatigue on attentional control and between-group differences

**Figure 6:**
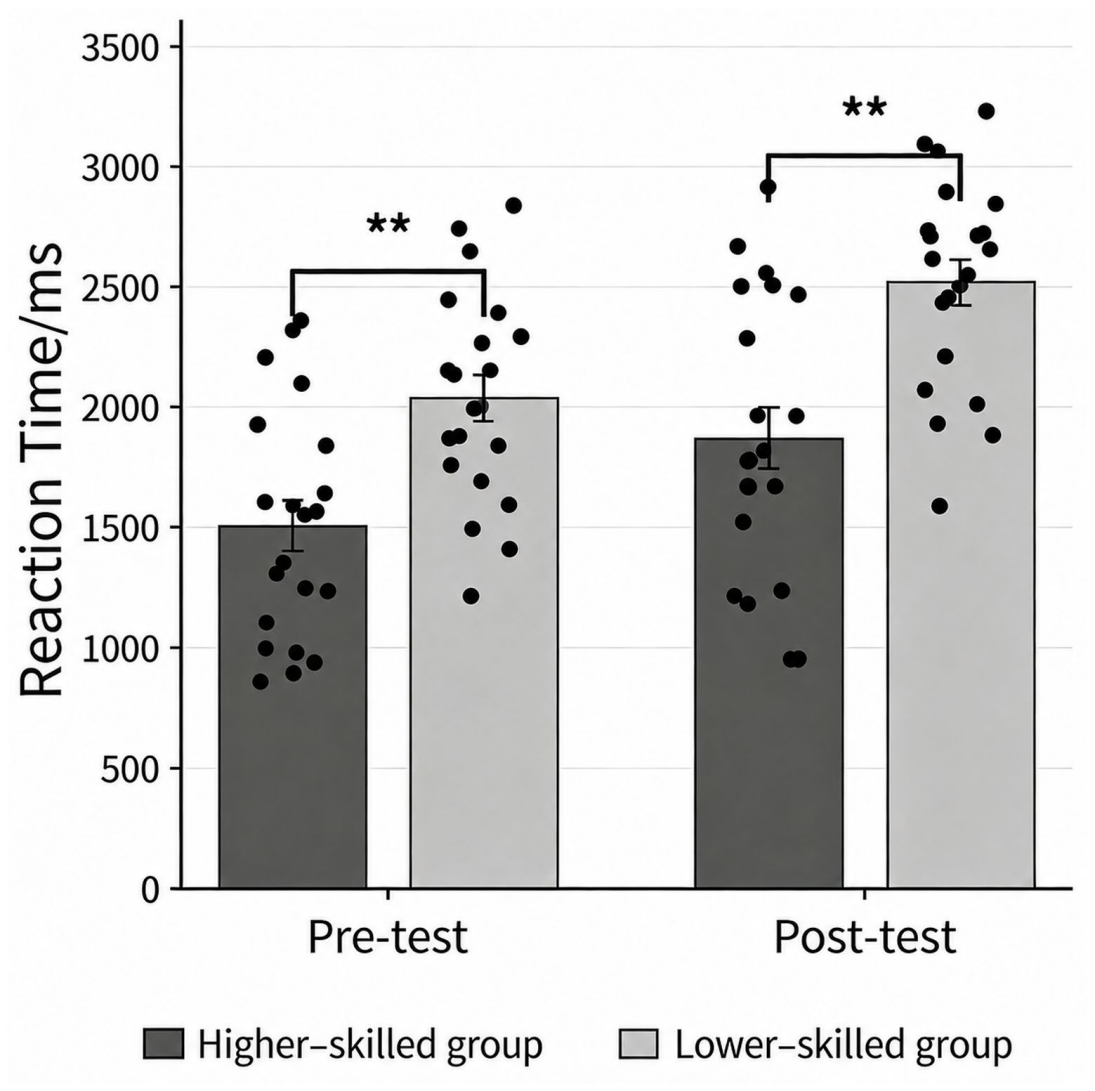
Comparison of flanker reaction time before and after fatigue across groups. *p < 0.05, **p < 0.01, ***p < 0.001.

**Figure 7:**
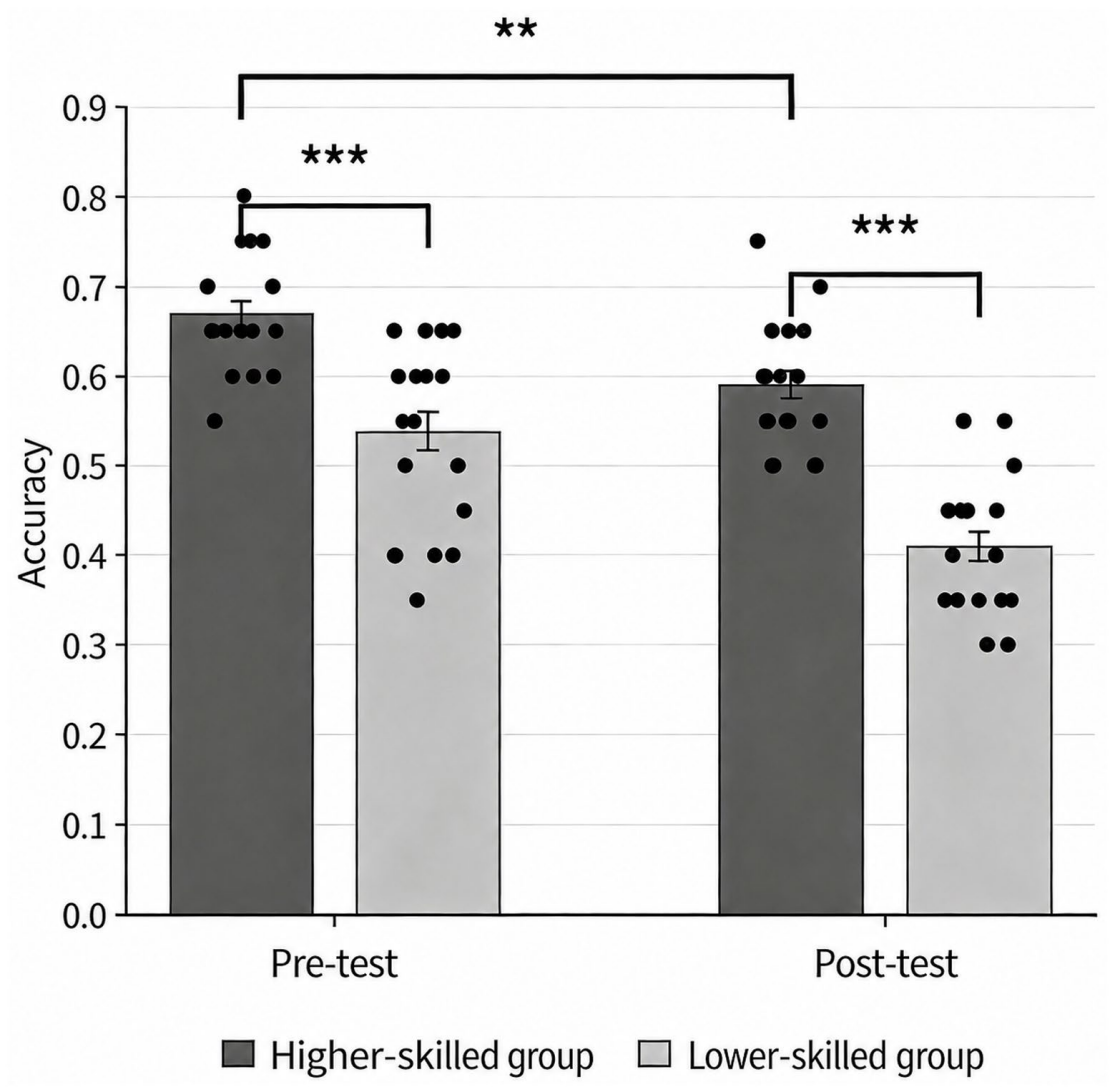
Comparison of flanker accuracy before and after fatigue across groups. *p < 0.05, **p < 0.01, ***p < 0.001.

Attentional control was indexed by the Flanker conflict effect and its pre-to-post change following fatigue induction (post − pre). First, response time and accuracy in the congruent and incongruent conditions were summarized using descriptive statistics (M ± SD); the results are reported in Table 7. A repeated-measures ANOVA was conducted with the conflict effect as the dependent variable and time and group as factors. For accuracy, the main effects of time and group, as well as the interaction, were not significant (*p* > 0.05), indicating that under the present conditions, mental fatigue did not significantly affect the accuracy conflict effect. For response time, the main effect of time was significant, *F*(1, 40) = 146.091, *p* < 0.001, η*_p_*^2^ = 0.785, 95% CI (0.647, 0.849), indicating substantially longer RTs after induction than before induction. The main effect of group was significant, *F*(1, 40) = 24.739, *p* < 0.001, η*_p_*^2^ = 0.382, 95% CI (0.149, 0.552), indicating that RTs were shorter in the higher-skilled group than in the lower-skilled group. The time × group interaction was significant, *F*(1, 40) = 4.326, *p* < 0.05, η*_p_*^2^ = 0.098, 95% CI (0.000, 0.284), indicating that the magnitude of the pre-to-post change in RT differed between groups. Follow-up simple effects analyses showed that RT increased significantly after mental fatigue induction in both groups (*p* < 0.01), with a more pronounced increase in the lower-skilled group than in the higher-skilled group (*p* < 0.01). These findings suggest that fatigue-related changes in RT-based attentional control differed as a function of skill level (see Figures 6, 7). The accuracy conflict effect did not differ significantly from pre- to post-induction, suggesting that accuracy-based indices were not sufficiently sensitive to fatigue-induced changes in attentional control under the present experimental conditions. In addition, overall accuracy in the Flanker task was relatively high, which may have restricted the variability of the accuracy conflict effect. According to attentional control theory, adverse psychological states typically impair processing efficiency first, as reflected in longer reaction times, whereas performance effectiveness indices, such as accuracy, may remain relatively stable because of insufficient task difficulty, response-strategy adjustment, or ceiling effects. Therefore, compared with the accuracy conflict effect, the RT conflict effect may provide a more sensitive indicator of fatigue-related changes in attentional control efficiency (Ozturker et al., 2025). Based on this theoretical rationale and the observed data pattern, the pre–post change in the Flanker RT conflict effect was used as the primary attentional control indicator in the subsequent mediation analyses, whereas the accuracy conflict effect was examined as a supplementary index.

**Table 7:** Descriptive statistics of Flanker conflict effects before and after mental fatigue in basketball players of different skill levels (M ± SD).

Group	*n*	Flanker conflict effect ACC (%)		Flanker conflict effect RT (ms)	
		Pre-fatigue	Post-fatigue	Pre-fatigue	Post-fatigue
Higher-skilled group	21	2.13 ± 3.41	2.09 ± 3.43	24.56 ± 6.38	49.18 ± 16.20
Lower-skilled group	21	2.24 ± 5.56	1.89 ± 5.47	34.90 ± 9.86	69.75 ± 16.10

Values are mean ± SD. ACC, accuracy; RT, reaction time.

### 3.4 Correlation analyses among subjective fatigue rating change, attentional control, and sport decision-making

Because group difference tests alone could not establish the relationships among the three variables, correlation analyses were conducted to further examine the associations among subjective fatigue rating change, attentional control, and sport decision-making (see Table 8). To reduce the potential confounding effect of skill-level differences on these associations, overall Pearson correlations were first computed, and partial correlations controlling for group membership were then calculated; the partial correlation results served as the primary basis for interpretation. The results showed that, after controlling for group membership, subjective fatigue rating change was significantly positively correlated with the reaction-time index of attentional control [*pr*(39) = 0.655, *p* < 0.001] and with sport decision-making reaction time [*pr*(39) = 0.805, *p* < 0.001]. These findings indicate that greater subjective fatigue rating change was associated with longer reaction times in both attentional control and sport decision-making. A significant positive correlation was also observed between attentional control reaction time and sport decision-making reaction time [*pr*(39) = 0.784, *p* < 0.001], indicating that longer reaction times in attentional control were associated with longer reaction times in sport decision-making. In addition, the correlation between subjective fatigue rating change and decision-making accuracy was not significant [*pr*(39) = 0.163, *p* = 0.310], nor was the correlation between attentional control reaction time and decision-making accuracy [*pr*(39) = 0.208, *p* = 0.192]. The overall Pearson correlations indicated that subjective fatigue rating change was significantly positively correlated with attentional control reaction time (*r* = 0.682, *p* < 0.001) and sport decision-making reaction time (*r* = 0.904, *p* < 0.001). Attentional control reaction time was significantly positively correlated with sport decision-making reaction time (*r* = 0.769, *p* < 0.001), whereas its correlations with decision-making accuracy were not significant (*p* > 0.05). The partial correlation results after controlling for group membership were consistent in direction with the overall Pearson correlations. Overall, the significant associations among the variables were primarily observed for reaction-time measures, whereas no significant linear relationships emerged for accuracy.

**Table 8:** Partial correlation matrix.

Variables	*M*	*SD*	1	2	3	4
Subjective fatigue rating change	39.02	11.20				
Attentional control (RT)	29.47	16.73	0.655***	1		
Sport decision-making accuracy	−0.10	0.06	0.163	0.208	1	
Sport decision-making (RT)	441.41	248.36	0.805***	0.784***	0.145	1

*Indicates *p* < 0.05, **indicates *p* < 0.01, ***indicates *p* < 0.001.

### 3.5 Mediation of attentional control and conditional process analyses

#### 3.5.1 Mediation analysis of mental fatigue, attentional control, and sport decision-making

**Figure 8:**
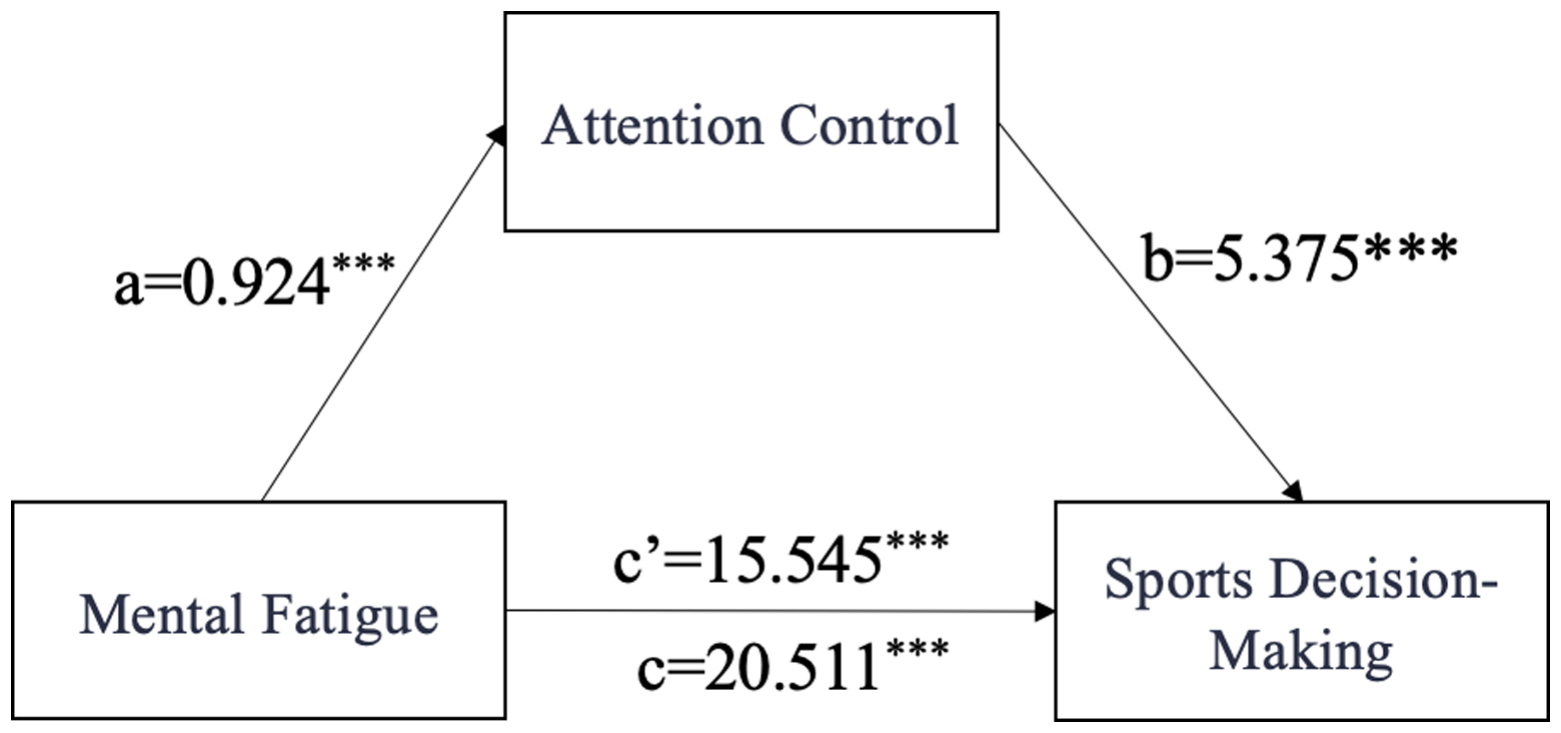
Mediation model of mental fatigue, attentional control, and sport decision-making.

PROCESS Model 4 (Preacher and Hayes, 2008) was used to test the mediating role of attentional control in the relationship between mental fatigue and sport decision-making performance, with skill level entered into the model as a covariate. The pre–post difference in decision-making reaction time was entered as the outcome variable, and the pre–post difference in the Flanker RT conflict effect was entered as the mediator. HC3 heteroscedasticity-robust standard errors were used, and bootstrap resampling (Bolin, 2014) with 5,000 iterations was conducted to estimate 95% confidence intervals. Detailed results are presented in Tables 9, 10. The results indicated that subjective fatigue rating change significantly and positively predicted the RT index of attentional control (*B* = 0.924, *t* = 3.505, *p* = 0.001). After subjective fatigue rating change and the RT index of attentional control were entered simultaneously into the model, the RT index of attentional control remained a significant positive predictor of sport decision-making performance (*B* = 5.375, *t* = 4.396, *p* < 0.001). The direct effect of subjective fatigue rating change on sport decision-making performance was significant (*B* = 15.545, *t* = 9.108, *p* < 0.001), and the total effect of subjective fatigue rating change on sport decision-making performance was also significant (*B* = 20.511, *t* = 11.096, *p* < 0.001). The bootstrap analysis revealed a significant indirect effect of subjective fatigue rating change on sport decision-making performance through the RT index of attentional control [95% BootCI (1.621, 8.513)]. From a descriptive perspective, the indirect effect accounted for approximately 24.2% of the total effect. It should be noted that the standard PROCESS output does not directly provide a bootstrap confidence interval for this proportion. Therefore, this proportion was reported only as a supplementary descriptive index of effect decomposition and was not used as an independent basis for statistical inference. The statistical significance of the mediation effect was determined primarily based on the bootstrap confidence interval for the indirect effect (see Figure 8).

**Table 9:** Regression results for the mediation model.

Dependent variable	Predictor	*B*	*SE*	*t*	*p*	95% CI
M	Constant	−10.535	8.587	−1.227	0.227	[−27.903, 6.834]
	X	0.924**	0.264	3.505	0.001	[0.391, 1.457]
	Group	7.540	5.194	1.452	0.155	[−2.965, 18.045]
Y	Constant	−282.186	37.974	−7.431	*p* < 0.001	[−359.060, −205.311]
	M	5.375***	1.223	4.396	*p* < 0.001	[2.900, 7.850]
	X	15.545***	1.707	9.108	*p* < 0.001	[12.090, 19.000]
	Group	−79.067*	29.817	−2.652	0.012	[−139.429, −18.705]

*Indicates *p* < 0.05, **indicates *p* < 0.01, ***indicates *p* < 0.001.

**Table 10:** Decomposition of total, direct, and indirect effects (Bootstrap).

Effect type	Effect size	BootSE	BootLLCI	BootULCI	Proportion of total effect
Total effect	20.511	1.846	16.753	24.223	100.0%
Direct effect	15.545	1.707	12.090	19.000	75.8%
Indirect effect	4.966	1.747	1.621	8.513	24.2%

#### 3.5.2 Moderating role of skill level

To examine whether skill level (group: 0 = higher-skilled, 1 = lower-skilled) moderated the indirect effect of mental fatigue on sport decision-making performance through attentional control, PROCESS v4.1 Model 7 was employed (HC3 heteroscedasticity-robust standard errors, bootstrap = 5,000, 95% CI). The results indicated that, with the RT index of attentional control as the dependent variable, subjective fatigue rating change significantly predicted the RT index of attentional control (*B* = 1.062, *t* = 3.106, *p* < 0.01), whereas the interaction between subjective fatigue rating change and group was not significant (*B* = 0.591, *t* = 0.908, *p* = 0.370), suggesting that skill level did not significantly moderate the effect of subjective fatigue rating change on attentional control. Further examination showed that the index of moderated mediation was not significant [2.508, 95% BootCI (−2.386, 7.560)], indicating that the indirect effect did not differ significantly between the higher-skilled and lower-skilled groups. However, the conditional indirect effects were significant in both groups: 3.190 for the higher-skilled group, 95% BootCI (0.179, 8.164), and 5.698 for the lower-skilled group, 95% BootCI (0.630, 11.507). Taken together, the mediation effect was present in both groups but did not vary significantly as a function of skill level. A further *post hoc* sensitivity analysis showed that, with the current sample size of *N* = 42, *α* = 0.05, and power (1 − *β*) = 0.80, the minimum detectable effect size for a single interaction term was approximately f^2^ = 0.197. This result suggests that the current sample was mainly powered to detect medium or larger interaction effects, whereas smaller moderation effects or conditional indirect effects may have gone undetected. Therefore, the nonsignificant moderated mediation effect observed in the present study should be interpreted with caution.

#### 3.5.3 Supplementary analyses

To examine the robustness of the mediation model, sport decision-making accuracy was used instead of sport decision-making reaction time as the dependent variable. A bootstrap mediation analysis was then conducted using Model 4 in PROCESS v4.1, with group membership entered as a covariate. The results indicated that subjective fatigue rating change significantly and positively predicted the RT index of attentional control (*B* = 0.924, *t* = 3.505, *p* < 0.01). However, after the RT index of attentional control and group membership were entered simultaneously into the model, the direct effect of subjective fatigue rating change on sport decision-making accuracy was not significant (*B* = 0.0002, *t* = 0.202, *p* = 0.841), and the RT index of attentional control likewise did not significantly predict sport decision-making accuracy (*B* = 0.0007, *t* = 0.966, *p* = 0.340). The bootstrap analysis further indicated that the indirect effect was 0.0006, with a 95% confidence interval of (−0.0009, 0.0019). Because the confidence interval included zero, the mediation effect was not statistically significant. It should be noted that, although group membership had a significant effect on sport decision-making accuracy (*B* = −0.0535, *t* = −3.020, *p* = 0.005), no significant indirect pathway was observed when accuracy served as the dependent variable.

Given the between-group differences in years of training, supplementary mediation analyses were also conducted with years of training and age entered as covariates. The results indicated that, after controlling for years of training and age, the indirect effect remained significant [bootstrap 95% CI (0.9841, 8.6069)], and the direct effect was also significant, suggesting that the mediation effect remained robust after controlling for training experience and age. To examine whether the treatment of change scores influenced the mediation results, an additional mediation analysis was conducted using residualized change scores instead of simple pre–post difference scores. The results showed that subjective fatigue rating change significantly and positively predicted residualized attentional control change, *B* = 1.019, SE = 0.153, *t* = 6.647, *p* < 0.001, 95% CI (0.709, 1.329). Residualized attentional control change also significantly and positively predicted basketball-specific decision RT change, *B* = 5.390, SE = 1.379, *t* = 3.909, *p* < 0.001, 95% CI (2.598, 8.182). The bootstrap results indicated that the indirect effect through residualized attentional control change was significant, Effect = 5.494, BootSE = 2.052, 95% BootCI (1.607, 9.774), suggesting that the main mediation findings were robust to the use of residualized change scores.

To further examine whether a speed–accuracy trade-off was present in basketball-specific decision-making performance after fatigue induction, basketball-specific decision RT change was calculated as post-test RT minus pre-test RT, and decision accuracy decline was calculated as pre-test ACC minus post-test ACC. A partial correlation analysis was then conducted while controlling for group. The results showed that basketball-specific decision RT change was not significantly correlated with decision accuracy decline, *pr*(39) = −0.156, *p* = 0.331. This finding indicates that the present study did not obtain direct behavioral evidence supporting a speed–accuracy trade-off. Therefore, the interpretation that athletes may maintain accuracy by slowing their responses should be considered cautiously.

## 4 Discussion

### 4.1 Effects of mental fatigue on sport decision-making in basketball players

Using an E-Prime-based basketball decision-making task, the present study systematically examined the effects of mental fatigue on sport decision-making performance in basketball players. The results showed that, following completion of the mental fatigue induction task, participants demonstrated an average increase of 23.93% in decision-making reaction time and an average decrease of 10.4% in decision-making accuracy. These findings support H1, which proposed that mental fatigue impairs sport decision-making performance in basketball players, and are consistent with previous evidence indicating that mental fatigue undermines cognitive functioning (Bian et al., 2025). In sporting contexts characterized by high uncertainty and high information load, the effects of mental fatigue on decision-making are more readily reflected in reduced processing efficiency (Chen et al., 2019). Mental fatigue was associated with increased time demands for cue extraction, situation matching, and response selection, thereby prolonging reaction time. At the same time, the repeated-measures analyses further indicated that decision-making accuracy also declined significantly following mental fatigue induction, suggesting that when cognitive load accumulates continuously and exceeds a certain threshold, mental fatigue not only slows decision-making speed but also compromises decision quality (Eysenck et al., 2023).

It should be noted that the individual-difference correlation analysis showed no significant linear association between subjective fatigue change and decision accuracy change. This finding does not necessarily contradict the significant decline in accuracy observed in the repeated-measures ANOVA. The repeated-measures ANOVA primarily tested systematic within-participant changes from pre- to post-induction, whereas the correlation analysis examined whether between-participant differences in the magnitude of fatigue change could explain between-participant differences in accuracy change. Thus, these two analyses relied on different sources of variance. One possible explanation is that some athletes may have attempted to maintain accuracy under fatigue by slowing their responses or increasing their response threshold. However, because the present study did not directly manipulate the speed–accuracy trade-off or estimate response thresholds using approaches such as diffusion modeling, this interpretation remains a *post hoc* explanation and requires further verification in future research (Wang et al., 2023). Therefore, from the perspective of the overall response pattern, the effects of mental fatigue on sport decision-making are more readily reflected in response speed, whereas changes in accuracy are more likely to be jointly modulated by compensatory mechanisms and individual differences (Boksem et al., 2006). Overall, both decision-making speed and accuracy declined significantly following mental fatigue induction. In future training practice, coaches should carefully structure training content and intensity; during competition, they should monitor athletes’ fatigue levels in real time and adjust playing time and substitution strategies appropriately.

### 4.2 Role of skill level in modulating the effects of mental fatigue

The findings indicated that susceptibility to mental fatigue differed significantly between higher-skilled and lower-skilled basketball players. In terms of decision-making accuracy under mental fatigue, the lower-skilled group declined from 53.8 to 40.9% (a decrease of 12.9 percentage points), whereas the higher-skilled group declined from 66.9 to 59.0% (a decrease of 7.9 percentage points). This between-group difference was significant (*p* < 0.001). This result is consistent with the view that higher-skilled athletes possess cognitive advantages (Cao et al., 2025), in that higher-skilled athletes tend to exhibit more efficient cognitive processing structures and more stable strategy repertoires in key-cue extraction, situation representation, and pattern recognition, and are therefore better able to maintain decision quality in complex game situations (Fu, 2025). These findings provide partial support for H2, which proposed that, compared with the lower-skilled group, the higher-skilled group demonstrated greater decision stability under mental fatigue. Notably, the interaction between time and group was significant for accuracy but not for reaction time. This pattern suggests that the slowing associated with mental fatigue may reflect a general processing cost, such that athletes at different skill levels all incur greater temporal costs under fatigue, whereas declines in accuracy appear to more clearly reflect differences in skill level (Yu and Li, 2025). Athletes in the higher-skilled group typically accumulate more extensive practice experience and therefore accumulate a richer store of relevant decision episodes in memory. When confronted with decision-making demands, they are able to retrieve more appropriate memory representations, which may contribute to faster reaction times and more efficient information processing than those observed in lower-skilled players (Mou et al., 2025). By contrast, because their cognitive control resources are more vulnerable to depletion, lower-skilled players may be more likely, under fatigue, to exhibit biases in situation recognition, insufficient filtering of goal-relevant information, and mismatches in response selection, thereby resulting in reduced decision accuracy (Qian, 2024). Taken together, an advantage in skill level does not imply complete immunity to mental fatigue; rather, it is more likely manifested as a protective effect on decision accuracy. Accordingly, training programs should place greater emphasis on helping lower-skilled players extract key cues and maintain decision stability under fatigue.

### 4.3 Effects of mental fatigue on attentional control in basketball players

The results indicated that, following mental fatigue induction, the RT conflict effect in the Flanker task increased significantly, whereas the accuracy conflict effect did not reach statistical significance. This suggests that, under fatigue, athletes required more time for conflict monitoring and interference inhibition, while task performance effectiveness was maintained to some extent (Kuroda et al., 2025). This finding supports H3, which proposed that mental fatigue is associated with impaired attentional control. This indicates that the effects of mental fatigue on attentional control are not manifested uniformly across all indices, but are more likely to emerge first at the level of processing efficiency. This pattern is consistent with the central prediction of attentional control theory, which proposes that under adverse psychological states, individuals must allocate additional cognitive resources to maintain goal-directed processing; consequently, impairment is more likely to be reflected in prolonged reaction time, whereas accuracy does not necessarily decline in parallel (Eysenck et al., 2007). In athletes, this may mean that, following mental fatigue induction, they continue to attempt to preserve task quality through additional effort, a higher response threshold, or slower responding, and are therefore more likely to exhibit a pattern of “slower but not markedly worse” performance in the Flanker task. By contrast, accuracy is more readily influenced by ceiling effects, task difficulty, and compensatory strategies, and may therefore be a relatively less sensitive indicator (Heuer and Wühr, 2023).

More importantly, this pattern, in which processing efficiency is impaired first while performance effectiveness is relatively preserved, may represent a critical cognitive precursor to subsequent deterioration in sport decision-making performance. In addition, the significant main effects of time and group, together with the significant time × group interaction for the RT conflict effect, suggest that fatigue-related increases in conflict cost varied as a function of skill level. Taken together with the findings for sport decision-making, this suggests that reduced attentional control efficiency may represent a key cognitive mechanism through which mental fatigue impairs decision-making performance. When attentional control efficiency declines, athletes must exert greater effort to extract key cues and suppress irrelevant information in video-based decision situations, thereby slowing subsequent situational judgment and response selection. These findings suggest that mental fatigue is more likely to impair the processing efficiency of conflict inhibition and task-set maintenance, rather than basic stimulus discrimination ability (Li et al., 2019). This also provides a more direct cognitive explanation for why mental fatigue primarily affects the reaction-time component of basketball decision-making.

### 4.4 Mediating role of attentional control

The results of the present study support the mediating role of attentional control in the relationship between mental fatigue and sport decision-making performance (H4), a finding with important theoretical implications. According to attentional control theory, as proposed by Eysenck and colleagues (Eysenck et al., 2007), anxiety weakens higher-order attentional control functions, thereby increasing the relative influence of stimulus-driven automatic processing on behavior. The present findings suggest that mental fatigue may produce a similar effect: under fatigue, athletes exhibited an increased Flanker interference effect and poorer sport decision-making performance, indicating that their attentional resources were more readily diverted by task-irrelevant information. This suggests that mental fatigue may weaken the attentional control processes normally used to suppress interference and maintain task goals, thereby making decision-making more dependent on stimulus-driven information. These findings are consistent with previous cognitive research. For example, Boksem et al. (2005) demonstrated, using ERP evidence, that mental fatigue reduces goal-directed top-down attentional control, resulting in behavioral patterns that are more strongly driven by external stimuli. Accordingly, under mental fatigue, athletes may find it more difficult to suppress irrelevant interference in the competitive environment in a timely manner and may therefore be more likely to make hasty or erroneous judgments. These findings extend the application of attentional control theory to sport psychology and further suggest that attentional control may represent an important target for intervention when seeking to counteract mental fatigue and maintain high-level decision-making performance.

Building on the mediation findings, the present findings revealed a relatively clear pattern of dissociation between processing efficiency and performance effectiveness (Eysenck et al., 2023). Specifically, in both the Flanker task and the sport decision-making task, reaction-time measures reflected the effects of mental fatigue more consistently than accuracy measures. This pattern can be interpreted in light of attentional control theory, particularly its distinction between processing efficiency and performance effectiveness (Eysenck et al., 2007). According to this theory, adverse psychological states first impair the efficiency of goal-directed control, which is initially manifested as slower information processing, increased interference-inhibition costs, and delayed response selection. By contrast, indicators of performance effectiveness, such as accuracy, may be partially preserved over the short term through additional effort, a higher response threshold, or strategically slowed responding (Eysenck et al., 2023). Accordingly, in the present study, the Flanker RT conflict effect and sport decision-making reaction time were more sensitive to changes induced by mental fatigue, whereas accuracy-based indices were relatively less stable. This does not mean that mental fatigue failed to affect decision-making; rather, it more likely indicates that mental fatigue first impairs processing efficiency rather than immediately disrupting behavioral effectiveness. This finding helps explain why, in basketball, performance under fatigue may initially become slower before becoming less accurate, and further suggests that relying solely on accuracy measures may underestimate the true impact of mental fatigue on sport decision-making.

However, the significant mediating effect observed in the overall sample does not necessarily indicate that this pathway operates in the same manner across athletes with different skill levels. For this reason, the present study further examined skill level as a potential boundary condition and conducted an exploratory test of this mediating process. The results indicated that the indirect effect was significant in both the higher-skilled and lower-skilled groups; however, the confidence interval for the index of moderated mediation included zero, suggesting that the magnitude of the indirect effect did not differ significantly across skill levels (thus, RQ5 was not supported). On the one hand, the sample size of the present study was sufficient for a preliminary examination of the main effects and mediation effects, but statistical power remained limited for detecting interaction effects and moderated mediation effects. On the other hand, attentional control was operationalized using difference scores for the conflict effect, which may have introduced some measurement error and thereby reduced the likelihood of detecting interaction and moderated mediation effects. Therefore, the absence of a significant moderating effect in the current study does not definitively rule out the possibility of such an effect.

On this basis, the present study further conducted covariate-controlled analyses and alternative outcome-variable analyses to examine the robustness of the above interpretation. After controlling for years of training and age, the indirect effect remained significant, indicating that the main findings were not entirely attributable to differences in training experience. However, the significant predictive effect of years of training on sport decision-making reaction time also suggests that long-term training experience may independently contribute to decision-making performance. Further robustness analyses indicated that when sport decision-making accuracy was used in place of reaction time as the outcome variable, the indirect effect did not reach significance. This result further supports the present interpretation of a dissociation between processing efficiency and performance effectiveness (Eysenck et al., 2007), suggesting that the effects of mental fatigue on sport decision-making are more likely to emerge first as a decline in processing efficiency rather than as an immediate change in accuracy. This finding is also consistent with prior evidence indicating that time-based measures are more sensitive than accuracy measures to changes in control efficiency (Eysenck et al., 2023). Given that, under conditions of high motivation or strong task engagement, athletes may tend to preserve accuracy at the expense of speed (Kucina et al., 2023), accuracy may be relatively less sensitive to mental fatigue. This is also consistent with attentional control theory, particularly its distinction between effectiveness and efficiency (Cocks et al., 2016). Taken together, these robustness analyses not only strengthen the present interpretation, but also suggest that future research on sport decision-making should report both speed and accuracy measures, with particular emphasis on temporal indices that are more sensitive to changes in control efficiency.

### 4.5 Limitations and future directions

The findings of the present study suggest that attentional control may be an important cognitive mechanism through which mental fatigue affects basketball-specific decision-making performance, and that this effect is primarily reflected in the dimension of processing efficiency. Nevertheless, several limitations should be acknowledged. First, the basketball-specific decision-making task was based on video simulation and keypress responses, which did not fully capture the physical movement, offensive–defensive interaction, and situational pressure of real-game contexts. Therefore, the ecological validity of the task could be further improved. Second, the sample consisted only of male basketball players. In addition, although the lower-skilled group had a lower competitive level than the higher-skilled group, these participants were still National Second-Class athletes who had received systematic training rather than truly inexperienced novices. Thus, the findings cannot be directly generalized to female athletes, truly inexperienced beginners, or recreational basketball participants. Third, the neutral-video control condition was used primarily for the VAS subjective fatigue manipulation check and was not fully incorporated into the main behavioral analyses of the Flanker task and the basketball-specific decision-making task. Therefore, time-on-task effects cannot be completely ruled out. Fourth, the Flanker task and the basketball-specific decision-making task were administered in a fixed order. Although this helped maintain procedural consistency, task order effects, practice effects, and the cumulative cognitive load induced by the Flanker task itself may still have influenced subsequent basketball-specific decision-making performance. Fifth, the moderated mediation analysis was exploratory. The *post hoc* sensitivity analysis indicated that the current sample was mainly powered to detect medium or larger interaction effects; therefore, the study may have been underpowered to detect smaller moderation effects. Accordingly, the absence of a significant moderated mediation effect should not be interpreted as evidence that skill level does not serve as a boundary condition. Finally, the present study used pre–post difference scores and residualized change scores to represent changes in the key variables. Although the residualized change-score analysis supported the main mediation findings, change-score indices may still be affected by measurement error. Moreover, VAS change primarily reflects subjective fatigue experience after mental fatigue induction rather than the full construct of mental fatigue. Future studies should increase sample size, adopt complete control conditions and counterbalanced task-order designs, and combine latent change score models, multilevel mediation models, heart rate variability, EEG, eye-tracking indices, or pupil diameter measures to more comprehensively examine the mechanisms through which mental fatigue affects basketball-specific decision-making.

## 5 Conclusion

Mental fatigue significantly impairs basketball-specific decision-making performance, primarily by prolonging decision reaction times and reducing accuracy. Skill-level differences were observed in both sport decision-making performance and attentional control, particularly in fatigue-related changes in decision accuracy and RT-based attentional control. Mechanistic analyses indicate that reduced attentional control partially mediates the relationship between mental fatigue and prolonged decision-making reaction time; however, this pathway is not robust for accuracy, suggesting that the mechanistic effects of mental fatigue are more strongly reflected in the speed-related dimension of decision-making.

## Data Availability

The original contributions presented in the study are included in the article/supplementary material, further inquiries can be directed to the corresponding author.
